# Supplementary material for: A Pan-Cancer Assessment of RB1/TP53 Co-Mutations
Source: Cancers (Basel). 2022 Aug 30;14(17):4199. doi: 10.3390/cancers14174199 (PMC9454436; doi:10.3390/cancers14174199)
Supplement: Supplementary file 1 [file cancers-14-04199-s001.zip › supp_figures.pdf]

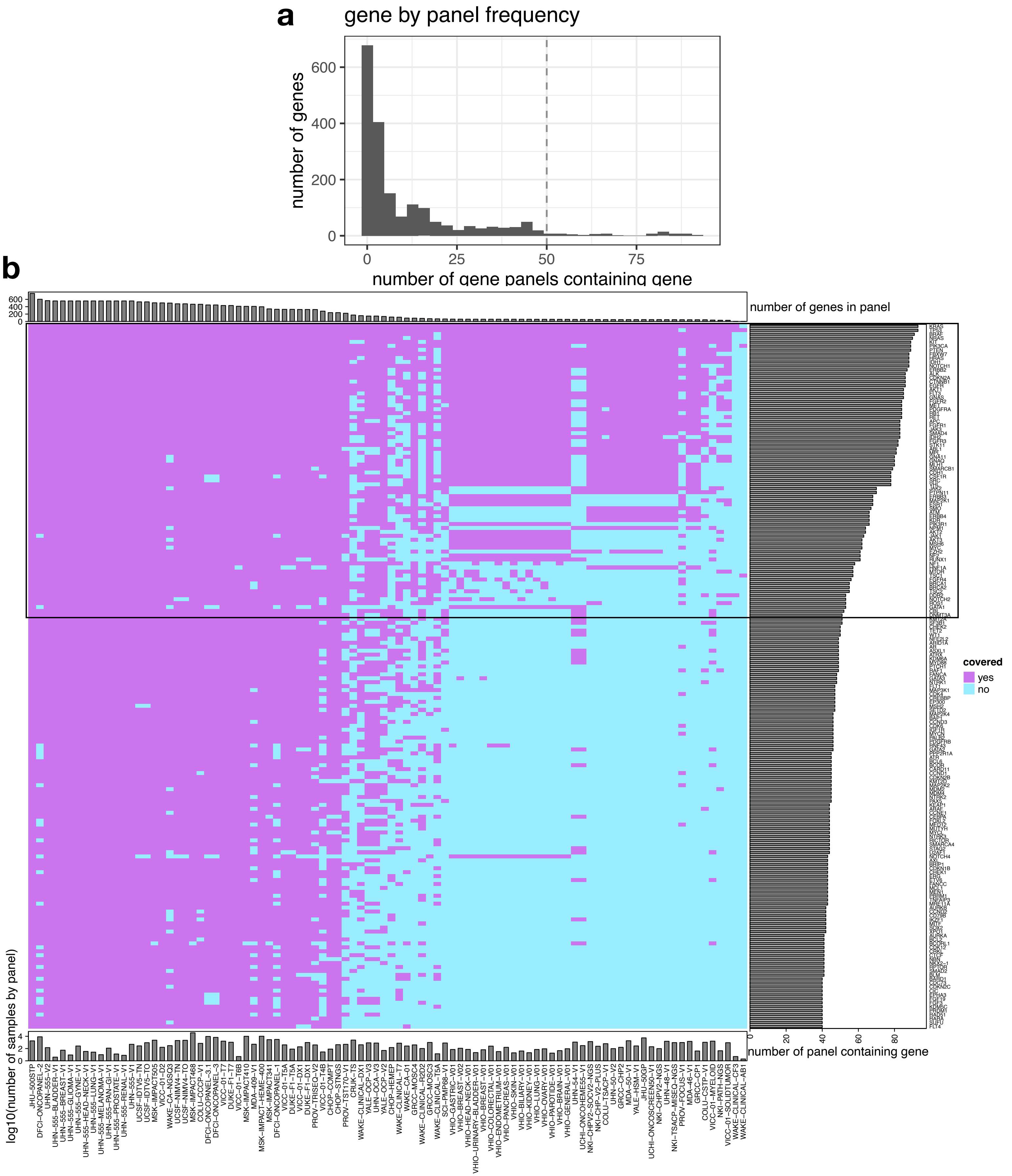

Figure S1. Selection of genes by gene panel coverage.

The 94 gene panels that feed data into AACR-GENIE have different gene coverage. To avoid bias in co-mutation analysis, we try to select genes that are covered by most panels. **a**. histogram shows the distribution of the number of genes as a function of the number of gene panels containing the gene. We selected 50 as a cut-off (drawn as a dashed line) for our study so that the gene we investigate is covered by over half of the panels. **b**. Assay coverage by gene and panel. This heatmap indicates whether a gene is (purple) or is not (cyan) covered in a specific assay. There are 178 genes covered by at least 40 panels in rows, and all 94 panels from the v11.0 AACR GENIE data in columns. The bar graph on top of the heatmap shows the number of genes covered by each panel. The bar graph on the right shows the number of panels covering each gene. The top part of the heatmap that contains the 74 genes selected by our custom cut-off is enclosed in a rectangle. The bar graph at the bottom shows the number of samples assessed by the assay.

Co-mutation check with AACR-GENIE v11.0 data | Review screening results | Adhoc analysis for a specific gene pair

Select gene 1: TP53

Select gene 2: RB1

Count in copy number alterations: no / yes

Display: table / scatter plot

SUBMIT

DOWNLOAD RESULT TABLE

Show 10 entries

Search:

number of samples with mutations in both genes

CANCER_TYPE_DETAILED	comutant	g1_mutant	g2_mutant	total	co.frac	g1.frac	g2.frac	g1_mut_given_g2_mut	g2_mut_given_g1_mut	co.pv	exclusive.pv	co.adj	exclusive.padj	sig.level	occurrence
Lung Adenocarcinoma	444	6140	562	13416	0.0331	0.4577	0.0419	0.0723	0.79	5.1e-61	1	1.5e-58	1	p.adj < .05	co-mutation
Bladder Urothelial Carcinoma	383	1365	478	2787	0.1374	0.4898	0.1715	0.2806	0.8013	2.7e-53	1	4.1e-51	1	p.adj < .05	co-mutation
Merkel Cell Carcinoma	79	97	85	317	0.2492	0.306	0.2681	0.8144	0.9294	4.5e-49	1	4.5e-47	1	p.adj < .05	co-mutation
Glioblastoma Multiforme	189	781	248	2266	0.0834	0.3447	0.1094	0.242	0.7621	2.9e-46	1	2.2e-44	1	p.adj < .05	co-mutation
Glioblastoma	123	639	156	1765	0.0697	0.362	0.0884	0.1925	0.7885	3.9e-30	1	2.4e-28	1	p.adj < .05	co-mutation
Breast Invasive Ductal Carcinoma	203	3816	275	8694	0.0233	0.4389	0.0316	0.0532	0.7382	1.5e-24	1	7.4e-23	1	p.adj < .05	co-mutation
Small Cell Lung Cancer	362	546	389	664	0.5452	0.8223	0.5858	0.663	0.9306	3.5e-18	1	1.5e-16	1	p.adj < .05	co-mutation
Pancreatic Neuroendocrine Tumor	20	78	24	522	0.0383	0.1494	0.046	0.2564	0.8333	2.1e-14	1	7.7e-13	1	p.adj < .05	co-mutation
Melanoma	38	415	72	2228	0.0171	0.1863	0.0323	0.0916	0.5278	3.3e-11	1	1.1e-9	1	p.adj < .05	co-mutation
Cancer of Unknown Primary	51	455	63	1082	0.0471	0.4205	0.0582	0.1121	0.8095	9e-11	1	2.7e-9	1	p.adj < .05	co-mutation

Showing 1 to 10 of 750 entries

Previous 1 2 3 4 5 … 75 Next

Figure S2. Screen shots from the "comut" web application for screening result review.
Users can specify a gene pair, and whether to include copy number alterations in mutation for analysis. The resulting table provides mutation counts and frequencies of single and co-mutations, as well p-values from Fisher's exact test assuming concurrent (co.pv) or mutually exclusive (exclusive.pv) co-mutations. P-values adjusted for multiple comparisons are also provided as "co.padj" or "exclusive.padj". Table is filterable and downloadable. Full name and explanation of the table header is provided in a tooltip upon mouseover. For example, in this snapshot, tooltip for "comutant" is displayed.

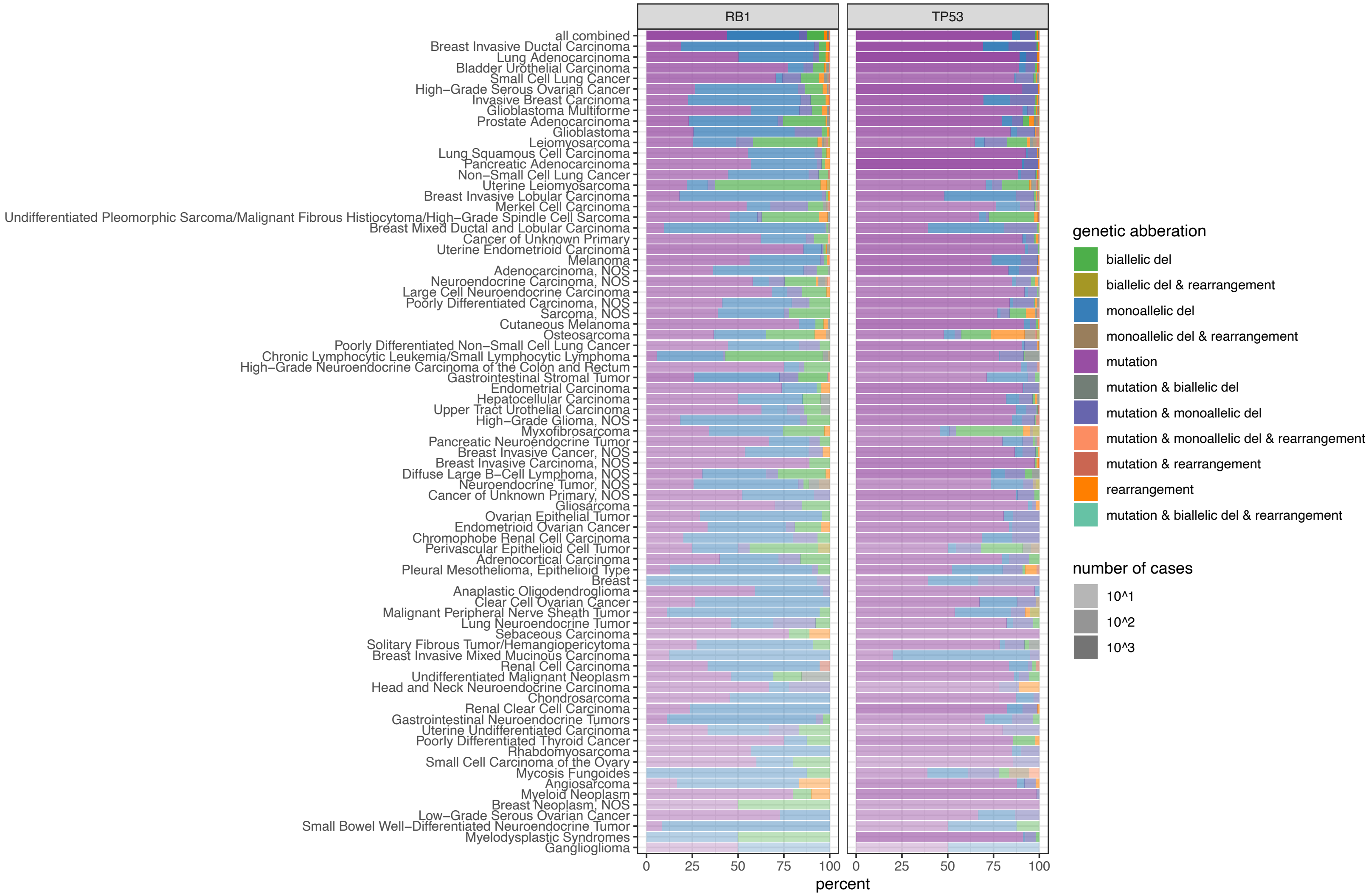

Figure S3. Genomic aberrations that lead to *RB1* and *TP53* loss by detailed cancer types.
Mutations, copy number alteration, and fusion data were integrated to determine *RB1* and *TP53* loss status. Distribution of the genomic alteration events on *RB1* and *TP53* were plotted for individual detailed cancer types that have significant enrichment of *RB1*/*TP53* co-mutation. Overall aberration distribution was also added on top of the plot as “all combined”. The detailed cancer types were ordered by the total number of co-mutated cases. The transparency of the stacked bars for each cancer type was determined by the number of cases with *RB1* or *TP53* mutations.

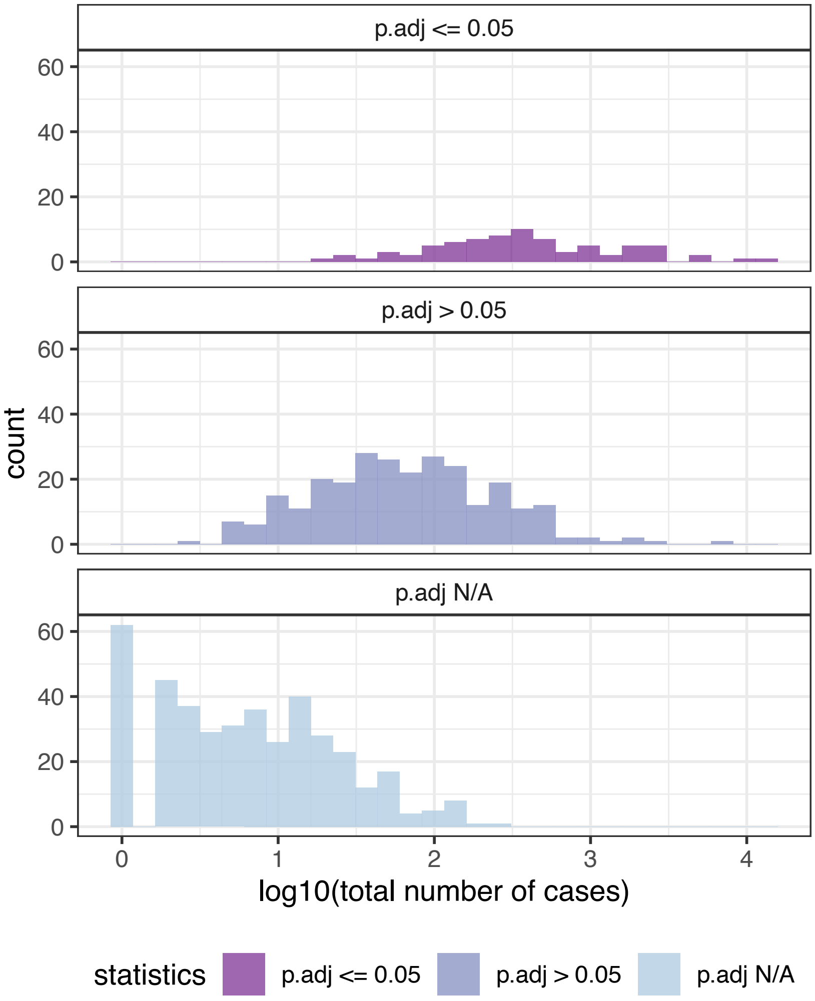

Figure S4. Co-mutation enrichment tests fail to reach statistical significance in cancer types with small sample sizes. The distribution of the total number of cases for different cancer types was compared among groups with different statistical significance from co-mutation enrichment tests. The cancer types with statistically insignificant *RB1*/*TP53* co-mutation enrichment tend to have smaller sample sizes.

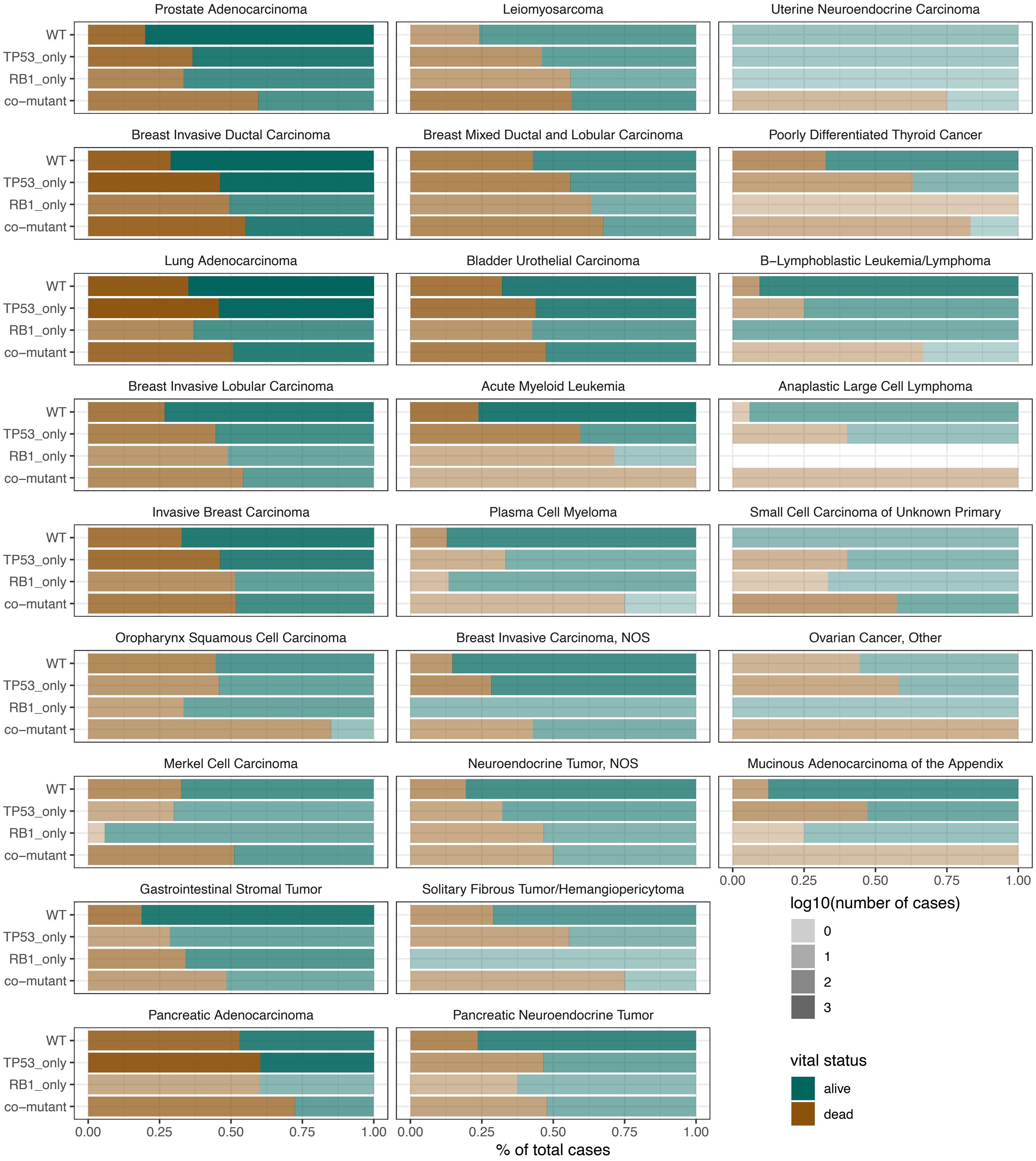

Figure S5. Death rate comparison across different RB1/TP53 oncogenotypes in different cancers. The fractions of dead and alive patients under the four *RB1/TP53* oncogenotypes are visualized for cancer types where *RB1/TP53* co-mutants were found to be enriched in the dead patients (see Figure 3a). The transparency of the bars represents the total number of cases under each oncogenotype. Note that the co-mutants almost always have the highest death rate in these different cancer types.

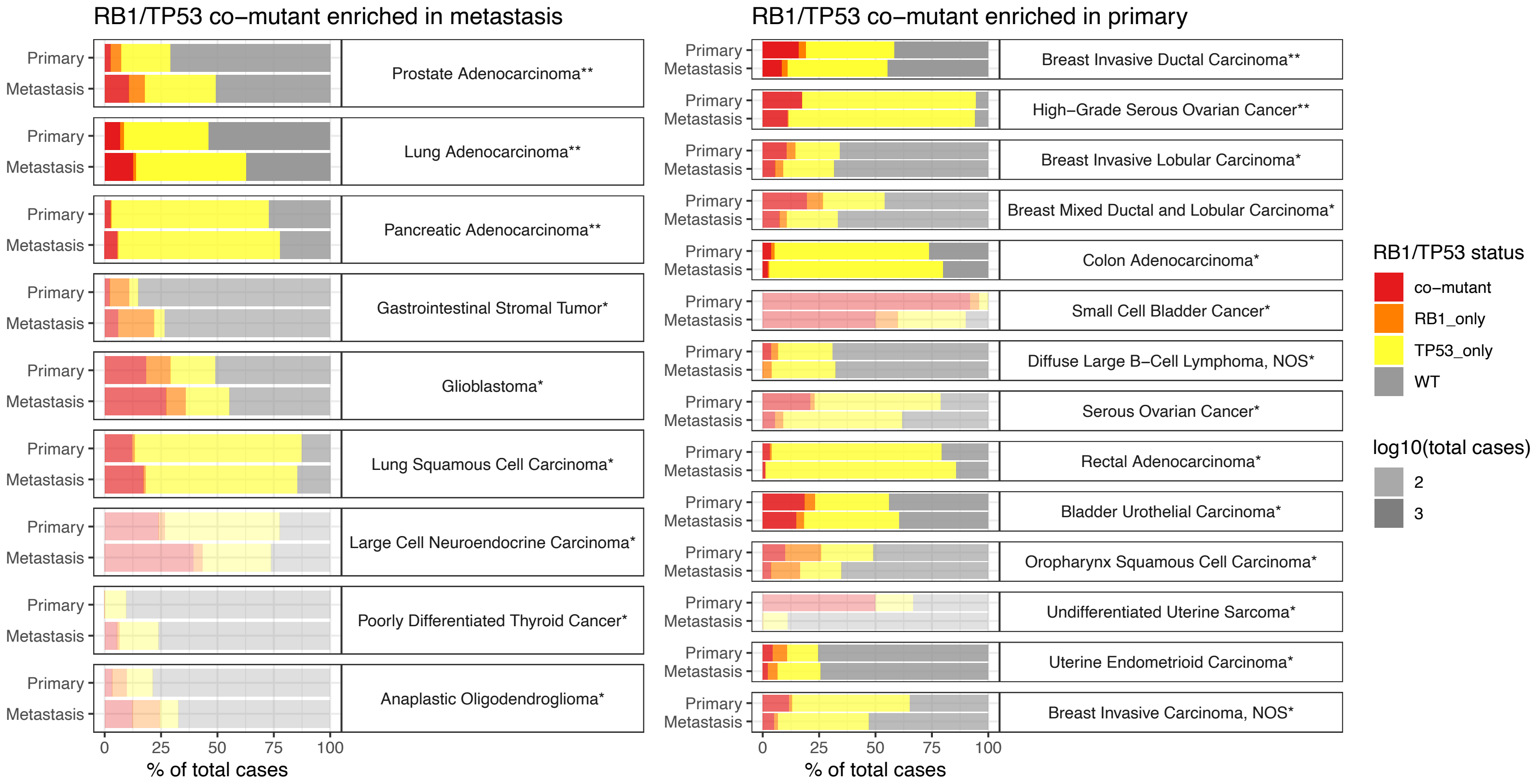

Figure S6. Association between *RB1/TP53* co-mutations and sample type.
Fisher's exact tests were performed to identify cancer types with RB1/TP53 co-mutations enriched in metastasis (left) or primary (right) tumors. All hits with a nominal p-value < 0.05 were plotted. The distribution of samples by *RB1/TP53* co-mutation status was visualized as stacked bar plots for each cancer type. P-values were denoted by asterisks (**, adjusted p-value < 0.05, *, nominal p-value < 0.05)

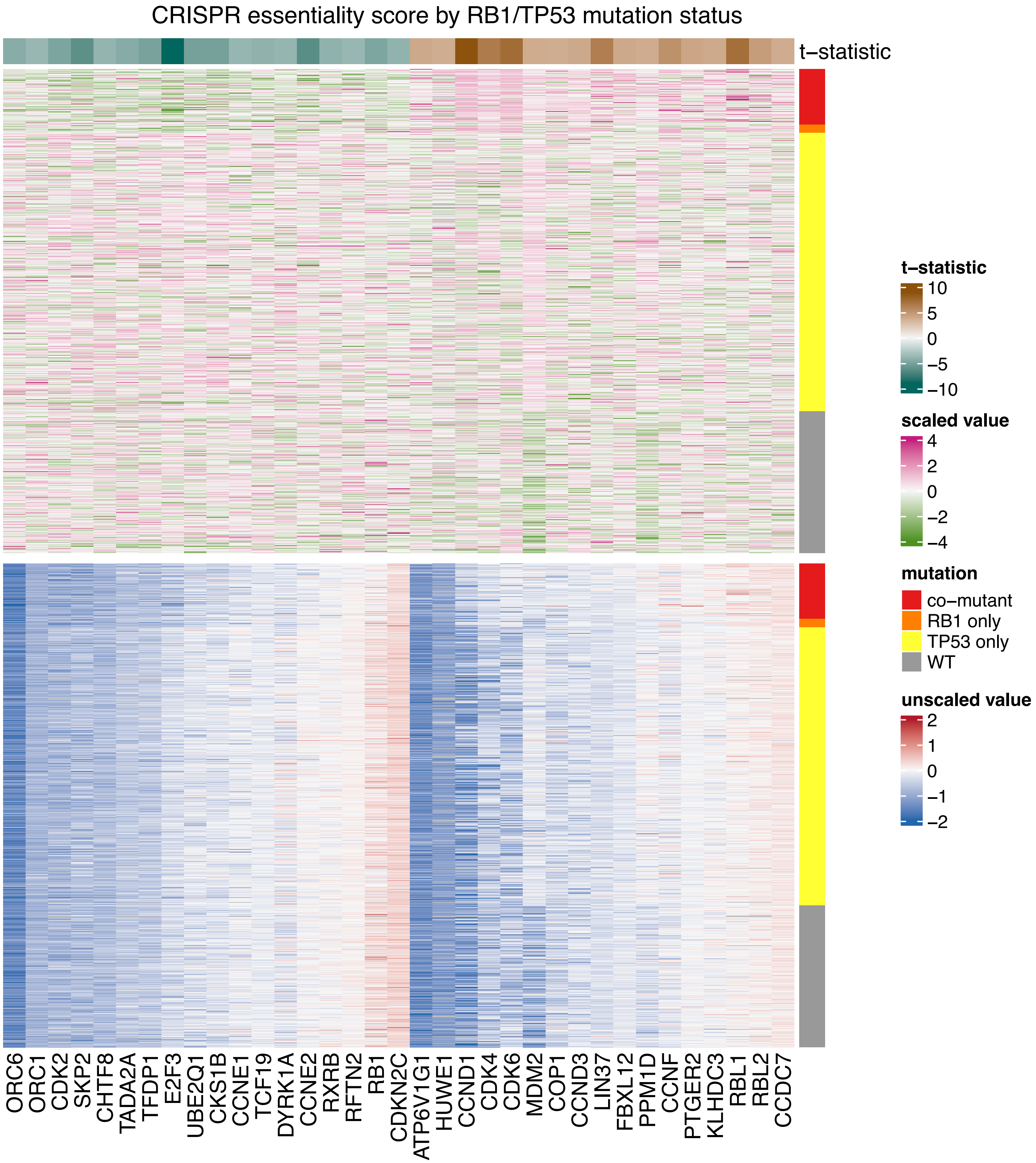

Figure S7. CRISPR essentiality scores by *RB1*/*TP53* mutation status for genes from differential CRISPR scores analysis
Scaled (z-transformed) gene essentiality scores are plotted in the top half of the heatmap to clarify the contrast between oncogenotypes whereas untransformed scores are plotted in the bottom half for better interpretability. t-statistic from lineage-adjusted linear model comparing co-mutant to WT cell lines are added to the top of the heatmap. Co-mutation status of the cell lines was annotated by the colored column right to the heatmap. Note that for the genes with negative t-values (left half), those with largely negative scores (blue, such as E2F3) are genes that have become more essential to the cell line with co-mutations. For genes with largely positive scores (red, such as RB1), the interpretation should be that cell lines with co-mutations did not gain additional growth advantage with CRISPR KO of such genes. For genes on the right side, those with predominantly negative scores (blue, such as CDK4), are genes that have become less essential to co-mutants, whereas those with predominantly positive scores (red, such as RBL1), are genes that could confer growth advantage when KO in the co-mutant background.
